# Progress and Perspectives in 2D Piezoelectric Materials for Piezotronics and Piezo‐Phototronics

**DOI:** 10.1002/advs.202411422

**Published:** 2025-03-12

**Authors:** Fengyi Pang, Pin Zhao, Hyeon Yeong Lee, Dae‐Jin Kim, Xiangchun Meng, Yong Soo Cho, Sang‐Woo Kim

**Affiliations:** ^1^ Department of Materials Science and Engineering Yonsei University Seoul 03722 Republic of Korea; ^2^ Division of Advanced Materials, Suzhou Institute of Nano‐Tech and Nano‐Bionics Chinese Academy of Sciences Suzhou 215123 P. R. China; ^3^ Department of Battery Engineering Yonsei University Seoul 03772 Republic of Korea

**Keywords:** 2D materials, biomedical applications, piezoelectric, piezotronics, self‐powered sensor

## Abstract

The emergence of two‐dimensional (2D) materials has catalyzed significant advancements in the fields of piezotronics and piezo‐phototronics, owing to their exceptional mechanical, electronic, and optical properties. This review provides a comprehensive examination of key 2D piezoelectric and piezo‐phototronic materials, including transition metal dichalcogenides, hexagonal boron nitride (h‐BN), and phosphorene, with an emphasis on their unique advantages and recent research progress. The underlying principles of piezotronics and piezo‐phototronics in 2D materials is discussed, focusing on the fundamental mechanisms which enable these phenomena. Additionally, it is analyzed factors affecting piezoelectric and piezo‐photoelectric properties, with a particular focus on the intrinsic piezoelectricity of 2D materials and the enhancement of out‐of‐plane polarization through various modulation techniques and materials engineering approaches. The potential applications of these materials are explored from piezoelectric nanogenerators to piezo‐phototronic devices and healthcare. This review addresses future challenges and opportunities, highlighting the transformative impact of 2D materials on the development of next‐generation electronic, optoelectronic, and biomedical devices.

## Graphical abstract

This review examines advancements in 2D materials, focusing on their applications in piezotronics and piezo‐phototronics. It discusses key materials like TMDs, h‐BN, and phosphorene, highlighting their unique mechanical, electronic, and optical properties. The review delves into the mechanisms of piezoelectricity, explores applications such as nanogenerators and biomedical devices, and describes the future and challenges in 3D integration of 2D materials.

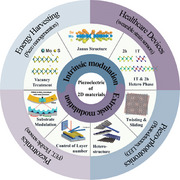

## 1 Introduction

Two‐dimensional (2D) materials have garnered extensive interest in recent years due to their unique physical and chemical properties that emerge at the atomic scale, which distinguish them from bulk materials. Structurally, 2D materials are characterized by strong in‐plane covalent bonds within layers and weak van der Waals interactions between layers. This configuration allows for precise control over layer number and stacking, leading to highly tunable properties in both mono‐ and few‐layer forms. For example, transition metal dichalcogenides (TMDCs) exhibit a range of electronic properties, from semiconducting to metallic or even superconducting, depending on composition, phase, and layer thickness, with bandgaps that transition from indirect to direct as the materials are thinned down to monolayer.^[^ ^1^, ^2^, ^3^ ^]^ Furthermore, 2D materials possess unique optical and electronic characteristics, such as tunable bandgaps and strong excitonic effects, which have paved the way for breakthroughs in photonics, optoelectronics, and catalysis.^[^ ^4^, ^5^, ^6^ ^]^ The flexibility in stacking TMDCs layers also enables the construction of lateral or vertical heterostructures, providing expanded functionality in solid‐state applications such as sensors, transistors, and energy devices.^[^ ^7^, ^8^ ^]^ Given these versatile properties, a promising application of 2D materials is in the realm of piezoelectricity, where their nanoscale dimensions and tunable mechanical properties offer potential for highly sensitive and efficient piezoelectric responses.

**Figure 1:**
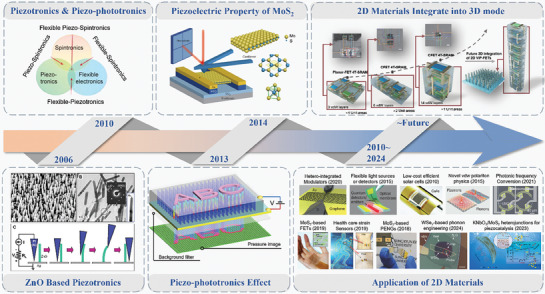
Evolution and research trends of 2D material‐based piezotronics and piezo‐phototronics. Reproduced with permission,^[^ ^9^ ^]^ Copyright 2006, American Association for the Advancement of Science. Reproduced with permission,^[^ ^37^ ^]^ Copyright 2010, Elsevier. Reproduced with permission,^[^ ^14^ ^]^ Copyright 2013, Springer Nature. Reproduced with permission,^[^ ^13^ ^]^ Copyright 2014, Springer Nature. Reproduced with permission,^[^ ^31^ ^]^ Copyright 2023, Springer Nature. Reproduced with permission,^[^ ^27^ ^]^ Copyright 2020, Springer Nature. Reproduced with permission,^[^ ^30^ ^]^ Copyright 2015, Springer Nature. Reproduced with permission,^[^ ^29^ ^]^ Copyright 2010, Springer Nature. Reproduced with permission,^[^ ^28^ ^]^ Copyright 2021, Springer Nature. Reproduced with permission,^[^ ^26^ ^]^ Copyright 2021, Springer Nature. Reproduced with permission,^[^ ^32^ ^]^ Copyright 2019, American Chemical Society. Reproduced with permission,^[^ ^33^ ^]^ Copyright 2019, American Chemical Society. Reproduced with permission,^[^ ^38^ ^]^ Copyright 2018, Wiley‐Blackwell. Reproduced with permission,^[^ ^35^ ^]^ Copyright 2024, Springer Nature. Reproduced with permission,^[^ ^34^ ^]^ Copyright 2023, Elsevier. Reproduced with permission,^[^ ^36^ ^]^ Copyright 2024, Springer Nature.

The development of piezoelectric nanogenerators (PENGs) utilizing ZnO nanowires, marked a significant milestone in the field of piezotronics and piezo‐phototronics.^[^ ^9^ ^]^ This work influenced for further exploration of piezoelectric effects in various nanomaterials.^[^ ^10^ ^]^ As shown in the **Figure** **1** in 2010, Wang et al. introduced the concepts of piezotronics and piezo‐phototronics,^[^ ^11^ ^]^ which leveraged the coupling of piezoelectric polarization with semiconductor properties to modulate electronic and optoelectronic processes at interfaces. The demonstration of ZnO nanowire‐based piezo‐phototronics in 2011 further solidified the foundation for these emerging fields.^[^ ^12^ ^]^ A significant breakthrough occurred in 2014 with the investigation of the piezoelectric effect in monolayer MoS_2_^[^ ^13^ ^]^ introducing 2D materials into the realms of piezotronics.^[^ ^14^, ^15^, ^16^, ^17^ ^]^ This discovery opened new possibilities for integrating 2D materials into advanced device architectures. Subsequent research has underscored the potential of 2D materials in photodiodes, photodetectors, low‐cost solar cells, and novel van der Waals heterostructures, highlighting their versatility in applications ranging from strain sensors to energy harvesting systems.^[^ ^18^, ^19^, ^20^, ^21^, ^22^, ^23^, ^24^ ^]^ For example, in the field of piezotronics the integration of 2D materials into piezoelectric and piezotronic devices has led to several innovative advancements. 2D materials based PENGs have demonstrated high efficiency in converting mechanical energy into electrical energy, which is crucial for self‐powered nanosystems.^[^ ^25^ ^]^ In the field of piezo‐phototronics, 2D materials have been utilized to enhance the performance of photodetectors and solar cells. By coupling the piezoelectric effect with photoexcitation, researchers have developed devices that exhibit improved charge separation and collection efficiency. This coupling effect between piezoelectricity and photoresponse has been particularly obvious in graphene, GaAs, hBN, MoS_2_ and WSe_2_‐based devices, which have shown remarkable improvements in light absorption and electrical output under mechanical strain.^[^ ^26^, ^27^ ^]^

In addition, since Wang et al. proposed piezotronics and piezo‐phototronics in 2010, 2D materials have produced a large number of fascinating application results in the past decade through heterostructure modulation pre‐strain modulation and material engineering modulation. 2D materials have been widely used in piezo‐phototronics fields: modulators, photodetectors, solar cells, polarion physics and photonic frequency conversion etc.^[^ ^26^, ^27^, ^28^, ^29^, ^30^, ^31^ ^]^ Moreover because 2D materials also have piezoelectric, catalytic and other properties, they play a huge role in field‐effect transistors (FETs), energy collection, phonon engineering, piezoelectric catalysis, health care and other fields.^[^ ^32^, ^33^, ^34^, ^35^ ^]^ In addition, the latest research by Guo et al. shows that the transition from 2D to 3D mode can be achieved through the heterostructure stacking of 2D materials. In the future, the application of 2D materials will be further explored and developed (**Figure** **1**).^[^ ^36^ ^]^

This review aims to provide a comprehensive overview of the recent advancements in the field of 2D piezoelectric and piezo‐phototronic materials, with a focus on their applications in PENGs, piezotronic devices, piezo‐phototronic systems and healthcare technologies. By studying the latest research findings, we focus on the potential of these materials to transform next‐generation electronic and optoelectronic devices. Additionally, the review also explores the fundamental mechanisms driving their piezoelectric properties and explores future directions for device integration. Through this analysis, we emphasize the pivotal role of 2D piezoelectric materials in the ongoing evolution of high‐performance, energy‐efficient technologies.

## 2 Synthesis Methods and Fundamental Principles of 2D Material

### 2.1 Synthesis Methods of 2D Materials

**Figure 2:**
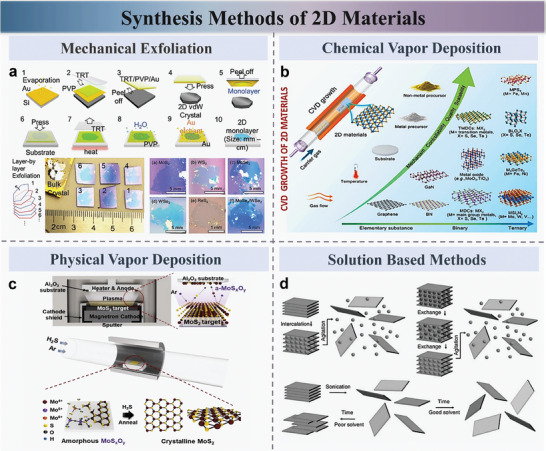
Synthesis methods of 2D materials. a) Schematic representation of the mechanical exfoliation process, illustrating the step‐by‐step procedure to isolate monolayer or few‐layer 2D materials from bulk crystals. b) Overview of the CVD process, showing the reaction of precursor gases on a substrate to form high‐quality 2D layered materials such as MoS_2_, WS_2_, and other TMDCs. c) Depiction of the PVD method, highlighting the use of a vapor‐solid process for the synthesis of 2D materials with controlled thickness and uniformity. d) Illustration of solution‐based methods, including exfoliation and intercalation, used for scalable production and functionalization of 2D nanosheets with precise layer control. Reproduced with permission,^[^ ^40^ ^]^ Copyright 2021, Elsevier. Reproduced with permission, ^[^ ^45^ ^]^ Copyright 2020, American Chemical Society. Reproduced with permission, ^[^ ^61^ ^]^ Copyright 2020, Welly. Reproduced with permission, ^[^ ^64^ ^]^ Copyright 2015, Elsevier.

Mechanical exfoliation: The mechanical exfoliation method is widely used to obtain atomically thin 2D materials from bulk crystals that are held together by weak van der Waals forces. This approach was initially employed in 2004 for graphene production.^[^ ^39^ ^]^ Mechanical exfoliation involves repeatedly applying adhesive tape to split bulk crystals, resulting in monolayer or few‐layer 2D materials. This straightforward cleavage technique has been successful in isolating various 2D layered materials such as graphene, hexagonal boron nitride (h‐BN), and TMDCs (**Figure** **2**a).^[^ ^40^, ^41^, ^42^, ^43^, ^44^ ^]^ The resulting materials are known for their high crystallinity and clean surfaces, making them ideal for basic research. However, its major limitation is the very low yield, which hinders scalability and broader application.

Chemical vapor deposition (CVD): CVD is a widely used technique to grow thin films on substrates via the chemical reaction of volatile precursors. This method enables the production of high‐quality 2D materials with controlled thickness and large‐scale uniformity, making it a valuable approach for applications in the electronics industry. As shown in Figure 2b, at the high temperatures and pressures, the precursor gases react at the substrate surface, supplying transition metal and chalcogen atoms that form thin‐layered MX_2_ materials, such as MoS_2_, WS_2_, MoSe_2_, and MoTe_2_.^[^ ^42^, ^45^, ^46^, ^47^, ^48^, ^49^, ^50^ ^]^ CVD synthesis offers a flexible platform for engineering 2D materials, as it allows for modifications like doping, alloying and heterostructure formation.^[^ ^51^, ^52^, ^53^, ^54^, ^55^, ^56^, ^57^, ^58^, ^59^ ^]^ These modifications enhance the tunability of electronic, optical, and magnetic properties, expanding the functional versatility of 2D TMDCs. CVD not only achieves high‐quality, large‐scalable 2D materials with consistent yield but also enables the construction of heterostructures with clean, atomically sharp interfaces. This makes CVD a promising technique for both foundational research and practical device integration.

Physical vapor deposition (PVD): PVD approach aims to obtain 2D materials by recrystallization of materials through a vapor‐solid process. Various 2D materials can be prepared by PVD process. Taking MoS_2_ as an example, Sohn et al. (Figure 2c).^[^ ^60^, ^61^ ^]^ reported the synthesis of layer‐controllable atomically‐thin MoS_2_ monolayer and few layers with this method. By using MoO_3_ seeds which deposited by magnetron sputtering as the precursor and sapphire as the substrates, MoS_2_ with the large‐scalable uniformity could be achieved under the post‐annealing process in H_2_S gas condition. By controlling the deposition time during the PVD process, the number of layers of MoS_2_ could be adjusted. However, the random nucleation of the crystals in PVD can make the layer thickness uneven, which requires more research efforts to resolve the issue.

Solution based methods: Solution‐phase techniques offer a promising approach for the scalable synthesis, functionalization, and integration of 2D TMDCs and nanosheets, facilitating extensive exploration of 2D materials across a range of innovative applications.^[^ ^62^, ^63^ ^]^ These methods are particularly effective for producing large‐scale 2D TMDCs and MXene flakes, such as MoS_2_, MoSe_2_, WS_2_, and WSe_2_, with precise control over the number of layers (Figure 2d).^[^ ^64^, ^65^, ^66^, ^67^ ^]^ Solution‐based approaches offer several advantages over other fabrication techniques for 2D nanosheets, including low‐cost precursors, scalability, ease of separation and classification, and excellent solubility. These properties enable diverse chemical functionalization opportunities, enhancing the electronic, mechanical, and chemical properties of the nanosheets when combined with other materials in solution. Moreover, the solution phase allows for the efficient transfer of 2D materials onto substrates, providing a practical pathway for further processing. However, solution‐phase methods do face challenges, such as the need for precise thickness control, potential contamination from solvents, and issues with purity, often necessitating further purification. While these techniques provide versatility, they can be less effective in achieving the high quality required for specific 2D materials, and some solvents may raise environmental concerns, suggesting a need for refinement in scaling up these methods.

### 2.2 Fundamental Principles of Piezotronics and Piezo‐Phototronics

Piezotronics and piezo‐phototronics are new and exciting fields that use the unique properties of piezoelectric materials to improve electronic and optoelectronic devices.^[^ ^68^, ^69^, ^70^ ^]^ In piezotronics, the key idea is to use the electric potential created by mechanical strain—called piezopotential—to control how electrical charges move at the interfaces of materials, like those between metal and semiconductor (M‐S) contacts or in p‐n junctions. Think of this piezopotential as a switch or a “gate” that can open or close to regulate electron flow.^[^ ^71^ ^]^ Similarly, piezo‐phototronics uses the piezopotential to affect the generation, separation, and transport of photogenerated carriers, therefore optimizing the performance of photodetectors, solar cells and piezo‐phototransistor.^[^ ^72^, ^73^, ^74^, ^75^ ^]^

**Figure 3:**
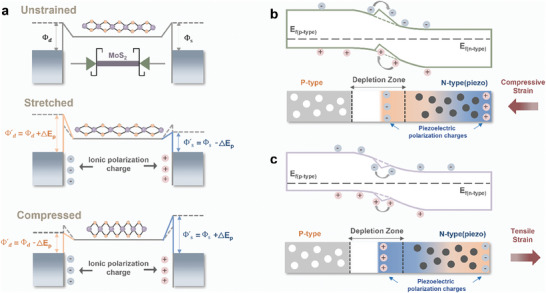
Strain effects on piezotronic and piezo‐phototronic properties in MoS_2_‐based devices. a) Schematic representation of how mechanical strain (unstrained, stretched, and compressed) affects the Schottky barrier height (SBH) at the metal‐semiconductor (M‐S) interface in piezotronic applications. Under tensile strain (stretched), ionic polarization charges generate a piezopotential that lowers the SBH, facilitating charge carrier transport. Compressive strain, on the other hand, induces opposite polarization, increasing the SBH and making charge carrier movement more difficult. b) Illustration of a Schottky junction under compressive strain in piezo‐phototronic applications, where positive piezoelectric polarization charges at the interface reduce the SBH, enhancing the generation, separation, and transport of photogenerated carriers. c) Depiction of a Schottky junction under tensile strain, where negative piezoelectric polarization charges increase the SBH, limiting carrier transport and reducing recombination. These modulations in piezopotential enable the dynamic control of carrier behavior, boosting the efficiency of photodetectors and solar cells through strain‐induced changes in the band structure and depletion zone width. Reproduced with permission,^[^ ^77^ ^]^ Copyright 2019, American Chemical Society.

In piezotronics, the M‐S contact is pivotal, with the piezopotential influencing the Schottky barrier height (SBH).^[^ ^76^, ^77^ ^]^ In simpler terms, when pressure is applied to a material like MoS_2_, it creates an electric field that changes the barrier height at the M‐S contact. If the material is squeezed (compressive strain), the SBH usually increases, making it harder for charges to pass through. If the material is stretched (tensile strain), the SBH decreases, allowing charges to move more easily (**Figure** **3**a). This control over the SBH is what allows piezotronics to create flexible sensors, actuators, and energy harvesters that work even when bent or pressed. Piezo‐phototronics, on the other hand, focuses on how the piezopotential affects carriers that are generated when light hits a material. This is important for devices like photodetectors and solar cells. In a Schottky junction, the piezopotential can change the band structure, making it easier for light‐generated charges to separate and move (Figure 3b,c). Under compressive strain, positive charges at the interface lower the SBH, helping carriers flow smoothly. If the material is under tensile strain, negative charges increase the SBH, which can slow down the flow and reduce recombination of these light‐generated carriers. In p‐n junctions, the piezopotential can change the width of the depletion layer—the region where electrons and holes separate. This affects how well the carriers can move, which is essential for the performance of photodetectors and solar cells.^[^ ^78^ ^]^ By controlling these processes, piezo‐phototronics can boost the efficiency of devices, paving the way for advanced energy‐harvesting and sensing technologies.

## 3 Factors Affecting Piezotronic and Piezo‐Phototronic Performance

### 3.1 Intrinsic Modulation of 2D Piezoelectricity

**Figure 4:**
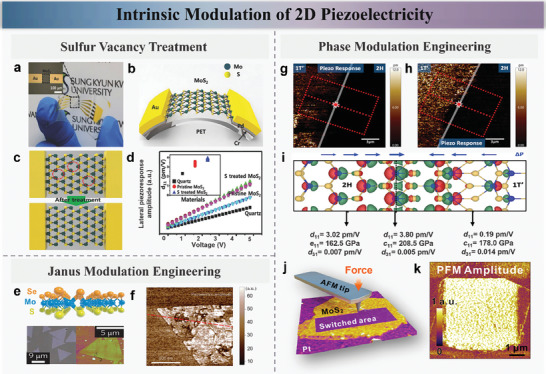
a,b) S vacancy‐passivated monolayer MoS_2_ PENG on flexible PET. c) Schematic of intrinsic S vacancies in pristine MoS_2_ and post‐treatment passivation. d) Lateral piezoelectric response of pristine MoS_2_, S‐treated MoS_2_, and α‐Quartz. e,f) Schematic and atomic force microscopy of Janus MoSSe structure. g,h) Piezoresponse at the 2H/1T′ MoTe_2_ interface. i) 1% lattice strain at the H–T′ interface induces electron density redistribution and enhances piezoelectricity and elasticity. j,k) Polarization switching in MoS_2_ by scanning probe microscopy and piezoelectric response in poled regions. Reproduced with permission,^[^ ^38^ ^]^ Copyright 2018, Wiley‐Blackwell. Reproduced with permission,^[^ ^85^ ^]^ Copyright 2017, Springer Nature. Reproduced with permission,^[^ ^86^ ^]^ Copyright 2022, Wiley‐Blackwell. Reproduced with permission,^[^ ^88^ ^]^ Copyright 2022, Springer Nature.

In the fields of piezotronics and piezo‐phototronics, the piezoelectric properties of materials are essential as they directly influence the energy conversion efficiency, sensing sensitivity, and signal processing capabilities of devices.^[^ ^79^, ^80^ ^]^ By manipulating the structure and defects of 2D materials such as MoS_2_ and MoTe_2_, researchers have identified several effective methods to enhance the piezoelectric coefficient. These methods include sulfur vacancy passivation, phase transition regulation, the introduction of Janus structures, and interface effect enhancement, all of which significantly improve the piezoelectric response.^[^ ^81^, ^82^, ^83^, ^84^ ^]^ These advancements not only boost the efficiency of energy harvesting and conversion but also enhance the sensitivity and reliability of sensing devices. As shown in **Figure** **4**a,b, Kim et al. demonstrated a sulfur vacancy passivated monolayer MoS_2_ PENG based on a flexible polyethylene terephthalate (PET) substrate.^[^ ^38^ ^]^ The study indicated that through the sulfur treatment process shown in Figure 4c, the sulfur vacancies on the MoS_2_ surface were effectively passivated, reducing carrier density and significantly preventing the screening effect of free carriers on piezoelectric polarization charges. The piezoelectric coefficient improved after post‐annealing showed in Figure 4d. The results showed that the output peak current and voltage of the sulfur‐treated monolayer MoS_2_ nanosheet PENG increased by 3 times (100 pA) and 2 times (22 mV) respectively, with the maximum power increasing nearly 10 times. Additionally, photoluminescence (PL) measurements indicated a significant enhancement in PL peak intensity post‐treatment, and Kelvin probe force microscopy (KPFM) showed an increase in the work function of MoS_2_. This study offers a novel method for enhancing the piezoelectric output performance of CVD‐grown MoS_2_, promising potential applications in stretchable and wearable electronic devices.

Beyond their in‐plane inversion asymmetry, additional degrees for spin manipulation can be introduced by breaking the out‐of‐plane mirror symmetry using external electric fields or asymmetric out‐of‐plane structural configurations. In this study, as shown in Figure 4e, Lu et al. report a synthetic strategy to fully replace the top‐layer sulfur atoms in MoS_2_ monolayer with selenium atoms, successfully growing Janus monolayer of MoSSe with broken out‐of‐plane structural symmetry. The Janus structure was directly confirmed through scanning transmission electron microscopy (TEM) and energy‐dependent X‐ray photoelectron spectroscopy, and the presence of vertical dipoles was demonstrated using second harmonic generation and piezoresponse force microscopy measurements (Figure 4f). The out‐of‐plane piezoelectricity in 2D monolayer introduces additional degrees for the design and motion control of practical nanoelectromechanical devices. Furthermore, this polar monolayer with enhanced Rashba spin–orbit interaction sets an important milestone for future spintronics.^[^ ^81^, ^85^ ^]^

Additionally, Puthirath et al. investigated a 2D in‐plane metal‐semiconductor junction made of 2H and 1T' phases of MoTe_2_.^[^ ^86^, ^87^ ^]^ Despite the weak piezoelectricity of each phase individually, a strong piezoelectric response was observed at the 2H–1T' junction using piezoresponse force microscopy. Experimental results and density functional theory calculations suggested that the amplified piezoelectric response at the junction is due to charge transfer across the semiconductor and metal junctions, forming dipoles and excess charge density (Figure 4g,h). As shown in Figure 4i, the piezoelectric coefficient *d*_11_ near the interface reached as high as 3.80 pm V^−1^, compared to 3.02 pm V^−1^ away from the interface.

Additionally, Lipatov et al. have experimentally observed stable room‐temperature out‐of‐plane polarization ordering in 2D MoS_2_ layers for the first time. Polarization switching was achieved through mechanical pressure applied by a scanning probe microscope (SPM), creating bi‐domain polarization states with distinct piezoelectric activity, second harmonic generation, surface potential, and conductivity (as shown in Figure 4j,k).^[^ ^88^ ^]^ The study indicates that the 1T′ phase of MoS_2_ belongs to a distorted trigonal structure, exhibiting spontaneous polarization confirmed by its P3m1 space‐group symmetry and theoretical modeling. Experimental results demonstrated that the piezoelectric coefficient *d*_33_ of 1T′ phase MoS_2_ is 0.7 ± 0.4 pm V^−1^ in the pristine region, while the mechanically poled region exhibits a significantly higher *d*_33_ value of 2.8 ± 0.4 pm V^−1^. The mechanically written domains are remarkably stable, suggesting potential applications of 1T′ phase MoS_2_ in flexible memory and electromechanical devices.

### 3.2 Extrinsic Modulation of 2D Piezoelectricity

**Figure 5:**
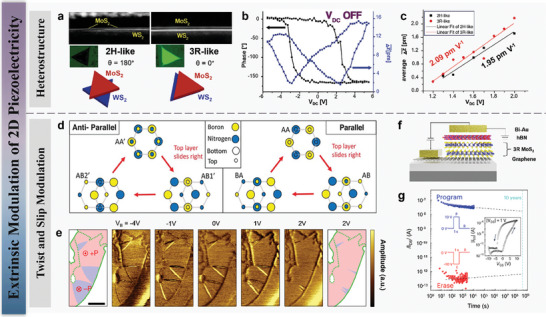
MoS_2_/WS_2_ heterostructure for ferroelectricity. a) TEM analysis of MoS_2_/WS_2_ heterostructures based pon different stacking (i. e. 2H, 3R). b) Phase and hysteresis loops of MoS_2_/WS_2_ c) Average piezoelectric coefficients of MoS_2_/WS_2_ heterojunctions. d) Top view of two‐layer stacking configurations of hBN. The top layer atoms are depicted as smaller circles for clarity. e) Lateral PFM measurement on gated MoSe_2_ device. The red and blue regions correspond to up and down domains, respectively. 3R stacking MoS_2_ for vertical ferroelectricity, f) schematic of the Bi/hBN/3R MoS_2_/graphene capacitor on a SiO_2_/Si substrate. g) Retention performance of the sliding‐ferroelectric 3R MoS_2_ FET. Reproduced with permission,^[^ ^109^ ^]^ Copyright 2022, American Association for the Advancement of Science. Reproduced with permission,^[^ ^110^ ^]^ Copyright 2021, American Association for the Advancement of Science. Reproduced with permission,^[^ ^103^ ^]^ Copyright 2022, Springer Nature. Reproduced with permission,^[^ ^107^ ^]^ Copyright 2023, Springer Nature.

In addition to the previous methods, it is well known that imparting 2D materials with ferroelectricity can also impart out‐of‐plane piezoelectricity. Recently, traditional 3D ferroelectric materials like PVDF and perovskites have become inadequate for extreme miniaturization. The loss of ferroelectricity in these materials as their size decreases necessitates the development of new ferroelectric materials.^[^ ^89^, ^90^ ^]^ Unlike traditional 3D ferroelectric materials, 2D materials with atomic‐scale thickness can achieve ultra‐thin device dimensions. Their smooth surfaces without dangling bonds allow the free construction of heterojunctions and integration with devices through van der Waals forces.^[^ ^91^, ^92^ ^]^ Starting from the magic‐angle that brought moiré patterns and superlattices to graphene, twisted 2D materials have been studied and reported one after that.^[^ ^93^, ^94^, ^95^ ^]^ This makes them highly promising for low‐power electronic devices, non‐volatile memories, photovoltaic and sensors.^[^ ^96^, ^97^, ^98^, ^99^, ^100^ ^]^ Studies have shown that techniques such as sliding and heterojunction formation can endow 2D materials with out‐of‐plane polarization.^[^ ^101^, ^102^, ^103^, ^104^, ^105^ ^]^ The principle is to destroy the spatial symmetry of the 2D material and form a dipole moment at the interface in the middle of the 2D material that can cause polarization reversal.^[^ ^106^, ^107^, ^108^ ^]^ As shown in the TEM of **Figure** **5**a,b, Lu et al. demonstrated the synthesis of untwisted, epitaxial MoS_2_/WS_2_ heterobilayers using a scalable one‐step CVD method.^[^ ^109^ ^]^ The experimental results revealed *d*_33_ piezoelectric constants ranging from 1.95 to 2.09 pm V^−1^, approximately six times higher than the natural out‐of‐plane piezoelectric constant of monolayer In_2_Se_3_ (Figure 5c). Moreover, by changing the polarization state of the MoS_2_/WS_2_ heterobilayers, they modulated the tunneling current in ferroelectric tunnel junction devices by about three orders of magnitude. These findings are consistent with density functional theory, indicating that ferroelectricity and out‐of‐plane piezoelectricity can be achieved through symmetry breaking and interlayer sliding without the need for twist angles or moiré domains. The one‐step CVD process enables large‐scale growth of such heterobilayers without requiring precision transfer methods or setups. This approach can be applied to other bottom‐up heterostructures, providing new insights for the development of ultra‐thin nanoelectronic devices and potentially playing an important role in future spintronics and optoelectronics.

In addition to heterojunction, twist and slip ferroelectricity is another way to give 2D materials ferroelectric properties. A large amount of high‐level experimental data and calculations have verified the feasibility of this method, giving new life to 2D materials. Shalom et al. reveals that when two h‐BN layers are stacked in a parallel, non‐centrosymmetric manner, a stable ferroelectric order emerges at the interface. This finding challenges the conventional understanding that layered diatomic crystals typically avoid internal polarization by forming a centrosymmetric lattice in their optimal van der Waals configuration.^[^ ^101^ ^]^ The researchers employed an experimental setup where h‐BN flakes, each consisting of a few layers, were stacked with a slight twist angle. This subtle adjustment led to the formation of alternating polarization domains at the interface.^[^ ^110^ ^]^ These domains, resulting from a lateral shift in the lattice positions, exhibited a non‐centrosymmetric stacking that allowed for the emergence of ferroelectricity. By applying a local electric field using a biased atomic force microscope (AFM) tip, they demonstrated the ability to reversibly switch the polarization by inducing lateral sliding of one layer relative to the other. Their findings are substantiated by a direct measurement of the voltage potential difference between the AB and BA stacking configurations, which ranged from 210 to 230 mV. This voltage difference confirms the presence of a robust out‐of‐plane polarization at the interface, consistent with theoretical predictions (Figure 5d). Further supporting their experimental results, density functional theory (DFT) calculations indicated a polarization per unit area of 0.33 Debye nm^−2^ for the AB‐stacked bilayer. The polarization was shown to be tunable by adding more layers or adjusting the lateral shifts between different stacking configurations. The ability to control polarization through twist angles and external electric fields suggests a novel way for designing memory devices and other applications where precise control of electronic properties at the atomic level is critical. The studies have the potential to impact the future of electronic materials and device engineering.

Jarillo‐Herrero et al. have made a notable contribution to the field of 2D materials by demonstrating interfacial ferroelectricity in rhombohedral‐stacked bilayers TMDCs.^[^ ^103^ ^]^ This research reveals that when two identical monolayer TMDCs are stacked in parallel with a slight twist angle, an out‐of‐plane polarization emerges due to the vertical alignment of atoms in the MX and XM stacking configurations. This discovery is particularly impactful as it introduces a new class of 2D ferroelectric semiconductors that combine the unique properties of TMDCs with ferroelectricity, a combination previously unobserved. Using piezoelectric force microscopy (PFM), the researchers observed distinct triangular domains within the bilayers structure, corresponding to MX and XM stackings. The strong contrast between these domains, particularly at the domain walls, indicates the presence of a spontaneous polarization that can be switched by applying an external electric field. As shown in the Figure 5e this polarization switching is visually represented in the PFM amplitude images, where domain walls shift in response to changes in the applied bottom gate voltage (V_B_). Specifically, at V_B_ = −4 V, both upward (+P) and downward (‐P) polarized domains coexist. As the voltage increases to 2 V, the downward domains shrink and nearly disappear, demonstrating the dynamic control of ferroelectric domains by an external electric field. Further experiments involved using graphene as a sensor to measure the built‐in interlayer potential of these bilayers TMDCs. The resistance measurements of graphene as a function of gate voltage revealed a clear hysteresis loop, confirming the ferroelectric nature of the material. The extracted interlayer potential, ≈55 mV, aligns well with theoretical predictions and is about half of that observed in parallel‐stacked bilayers h‐BN. This research not only expands the family of 2D ferroelectrics but also opens new possibilities for integrating ferroelectricity with the rich electronic and optical properties of TMDCs. The ability to control electronic conduction and optical responses via ferroelectric polarization in these materials could lead to novel applications in non‐volatile memory devices and beyond.

Lan et al. conducted a study on the development of ferroelectric transistors using rhombohedral‐stacked molybdenum disulfide (3R MoS_2_) to achieve switchable polarization, essential for non‐volatile memory devices.^[^ ^107^ ^]^ The research addresses the challenges of realizing switchable electric polarization in epitaxial MoS_2_, where conventional approaches were limited by the lack of mobile domain boundaries. The team employed a shear‐transformation mechanism in 3R MoS_2_, enabling the creation of ferroelectric domains with a switching field as low as 0.036 V/nm—significantly lower than traditional thin‐film ferroelectrics. These ferroelectric FETs (Fe‐FETs) demonstrated a notable memory window of 7 V and maintained performance over 10 000 cycles, with data retention times exceeding 10 000 s, highlighting the potential for low‐power, high‐density memory storage. The accompanying figure illustrates the long‐term stability and reliability of the Fe‐FETs. The retention tests show distinct current levels for the programmed state (blue) and erased state (red) over time, confirming the device's non‐volatile memory capabilities. The graph displays the drain current (I_DS_) as a function of time, with the program and erase operations defined by ±10 V gate pulses. The inset graph further reveals the hysteresis behavior of the Fe‐FETs, indicating robust polarization switching, with clear separation between the program and erase states over extended cycles (Figure 5f,g). This evidence supports the reliability and durability of the 3R MoS_2_ based Fe‐FETs, marking them as promising candidates for future memory applications.

**Figure 6:**
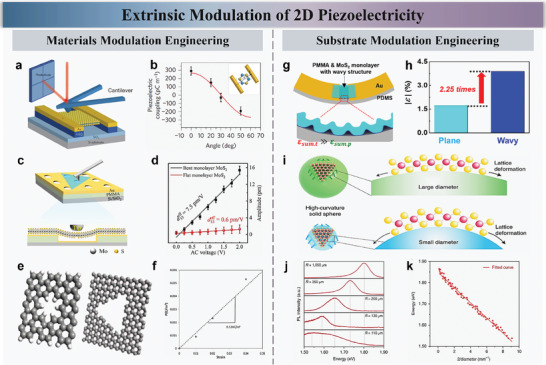
Materials modulation engineering method. a) In‐plane piezoelectric stress with AFM probe, the MoS_2_ film was suspended on SiO_2_ substrate. b) PFM analysis of the piezoelectric coupling effect at varying angles. The measured piezoelectric coupling strength follows the cos 3θ (as shown in the inset) dependence (red curve) predicted by the crystal's three‐fold symmetry. c) Schematic of the experimental setup with the AFM and a magnified side view of suspended MoS_2_. d) Average piezoresponse amplitude of bent and flat monolayer MoS_2_ as a function of the applied AC voltage. e,f) Inducing piezoelectricity in graphene by introducing holes with specific symmetries, showing the hole structures and their piezoelectric coefficients. Substrate modulation engineering method. g) Method to increase strain in MoS_2_ monolayers using wavy structures on pre‐stretched PDMS. h) As the tensile strain on the substrate doubles, a corresponding pre‐strain is induced in the MoS_2_. i) Schematic of sphere diameter engineering (SDE) technique for tuning the bandgap of 2D materials. j,k) PL measurements and energy‐diameter relationship showing bandgap tuning of MoS_2_ by glass spheres of different curvatures. Reproduced with permission,^[^ ^13^ ^]^ Copyright 2015, Springer Nature. Reproduced with permission,^[^ ^111^ ^]^ Copyright 2019, Wiley‐VCH Verlag. Reproduced with permission,^[^ ^112^ ^]^ Copyright 2012, American Institute of Physics. Reproduced with permission,^[^ ^114^ ^]^ Copyright 2023, American Chemical Society. Reproduced with permission,^[^ ^115^ ^]^ Copyright 2020, Springer Nature.

Zhang et al. tested the piezoelectric properties of MoS_2_ monolayer for the first time through experimental methods, which provided direction for subsequent piezoelectric research on MoS_2_.^[^ ^13^ ^]^ The research demonstrates that monolayer MoS_2_ exhibits piezoelectric properties only when the number of layers is odd, due to the breaking of inversion symmetry, while even‐layered structures show no piezoelectric response. This study not only confirms the theoretical predictions about piezoelectricity in 2D materials but also provides experimental evidence free from substrate effects, which often complicate such measurements. The researchers employed an innovative approach by using nano‐indentation and a lateral electric field in an atomic force microscope to measure the piezoelectric response (**Figure** **6**a). The results show in the Figure 6b revealed a piezoelectric coefficient of *e*_11_  =  2.9 × 10^−10^ C m^−1^, a value comparable to traditional bulk piezoelectric materials such as ZnO. Moreover, the study identified the angular dependence of the piezoelectric response, correlating it with the crystal's three‐fold symmetry. This angular dependence allowed the researchers to determine the absolute orientation of the 2D crystal, a significant step forward for low‐power logic switches and ultrasensitive sensors. Since then, there have been many articles on tuning the piezoelectric properties of 2D materials through materials engineering.

Hu et al. investigated the out‐of‐plane piezoelectricity in van der Waals layered materials, specifically focusing on MoS_2_ and InSe, demonstrating that this effect is significantly enhanced by flexoelectricity when the materials are free‐standing and curved.^[^ ^111^ ^]^ The study revealed that monolayer MoS_2_ exhibited an impressive effective out‐of‐plane piezoelectric coefficient *d*_33_ of ≈7.5 pm V^−1^, while few‐layered InSe reached up to 21.9 pm V^−1^. These findings underscore the potential of flexoelectricity in enhancing piezoelectric responses, especially in nanoscale applications. The authors utilized piezoresponse force microscopy (PFM) to measure these properties, carefully avoiding substrate‐induced artifacts, and found that the piezoelectric response increased with higher curvature and decreased with the number of layers. Figure 6c illustrates the experimental setup where an AFM tip applies an AC voltage to a free‐standing monolayer MoS_2_ membrane, leading to the measurement of piezoresponse. As shown in Figure 6d, the data clearly demonstrate that the bent MoS_2_ configuration yields a significantly higher piezoelectric response *d*_33_ = 7.5 pm V^−1^ compared to the flat configuration *d*_33_ = 0.6 pm V^−1^. In Figure 6e,f, Sharma et al. demonstrated that introducing holes with specific symmetries into graphene can induce piezoelectricity under applied strain by ensuring a semiconducting nature.^[^ ^112^ ^]^ The study found that porous graphene sheets with specific hole designs achieved a piezoelectric coefficient of 0.124 C m^2^, which is 72% of quartz or 36% of boron nitride nanotubes. This discovery opens new avenues for the multifunctional material design of graphene, particularly in piezotronic and microbial inactivation.^[^ ^113^ ^]^

Moreover, modifying the substrate to introduce stress into 2D materials can not only tune their piezoelectric properties but also improve their optoelectronic response by adjusting the bandgap, which is significant in piezotronics and piezo‐phototronics. Sohn et al. innovatively enhanced the output performance of PENGs based on monolayer MoS_2_ using a dual strain concentration method.^[^ ^114^ ^]^ As shown in Figure 6g,h, introducing wavy structures on pre‐stretched PDMS significantly increases the strain in the MoS_2_ monolayer, which is further amplified through bending. This method achieved a piezoelectric output voltage of 580 mV and a current of 47.5 nA, the highest reported values for 2D material‐based PENGs. These results, validated by finite element method simulations, demonstrate an improvement of ≈2.25 orders of magnitude over previous PENGs. As shown in Figure 6i, Zeng et al. proposed a sphere diameter engineering (SDE) technique to tune the bandgap of 2D materials.^[^ ^115^ ^]^ By adjusting the diameter of the supporting glass sphere, the bandgap of MoS_2_ can be precisely and continuously tuned within a range of 360 meV, as shown in Figure 6j,k. The experiments demonstrated an ideal linear relationship between the bandgap of MoS_2_ crystals and the sphere diameter. More importantly, both an increase and decrease in bandgap can be achieved by creating positive or negative curvature.

## 4 Applications

2D materials TMDCs being typical piezoelectric materials, there are many fascinating materials that also have excellent piezoelectric properties. For example, Group IV monochalcogenides such as GeSe and SnS exhibit remarkable piezoelectric properties due to their unique puckered crystal structures. Fei et al. reported that monolayer SnS and GeSe have piezoelectric coefficients that can reach values significantly higher than typical 2D materials like MoS_2_, with coefficients up to 16.1 pm V^−1^ for SnS and 10.2 pm V^−1^ for GeSe.^[^ ^116^ ^]^ These properties are due to their anisotropic in‐plane structure, enabling efficient energy harvesting and oxygen evolution reaction.^[^ ^117^, ^118^ ^]^ Moreover, Group III‐V Compounds of GaP and InP are known for their utility in electronic and optoelectronic devices, and they also possess notable piezoelectric properties in nanostructured forms.^[^ ^119^, ^120^ ^]^ Beya‐Wakata et al. conducted first‐principles calculations and showed that GaP exhibits a piezoelectric coefficient of ≈0.6 pm V^−1^. Although these values are lower compared to materials like MoS_2_, their potential for integration in strain‐tunable devices due to their compatibility with established semiconductor processes makes them valuable.^[^ ^121^ ^]^ In experimental studies, Lehmann et al. observed piezoelectric responses in nanowires of GaP, highlighting their application in nanoscale mechanical energy conversion.^[^ ^122^, ^123^ ^]^

In addition, CuInP_2_S_6_ (CIPS) has been demonstrated to have a strong out‐of‐plane piezoelectric response, distinguishing it among 2D materials. Hao et al. reported a high *d*_33_ piezoelectric coefficient of 17.4 pm V^−1^ for CIPS, surpassing many other 2D piezoelectric materials.^[^ ^124^, ^125^ ^]^ This material also exhibits robust room‐temperature stability, making it a promising candidate for nanoscale piezoelectric nanogenerators and devices integrated with silicon‐based technologies. CIPS also discovered that ferroelectricity has advantages over traditional polymer ferroelectric materials such as PVDF and PDMS, such as atomic‐level thickness and a clean surface without dangling bonds, and is expected to be used in e‐skin and non‐volatile memory.^[^ ^124^, ^126^, ^127^, ^128^, ^129^ ^]^ Additional, α‐In_2_Se_3_: Zhou et al. provided experimental evidence of both in‐plane and out‐of‐plane piezoelectricity in α‐In_2_Se_3_, with its piezoelectric coefficient ranging from 0.34 pm V^−1^ in monolayers to 5.6 pm V^−1^ in bulk. The noncentrosymmetric *R*3*m* structure of α‐In_2_Se_3_ facilitates multidirectional piezoelectric responses, making it suitable for applications in flexible nanogenerators and strain sensor.^[^ ^130^, ^131^, ^132^ ^]^ Xue et al. demonstrated that this material's piezoelectric properties can be leveraged for dual‐directional energy conversion, offering versatile applications in adaptive electronics.^[^ ^133^ ^]^ Although other materials such as GeSe, InP, and CIPS exhibit strong advantages in piezoelectric and physical properties, TMDCs hold unique benefits for practical applications. TMDCs not only excel in tunable bandgaps and piezoelectric responses but can also have their properties enhanced through engineering approaches like doping and heterostructure formation, thereby expanding their applications in piezotronic and optoelectronic devices. Notably, their excellent biocompatibility and non‐toxic nature make them highly promising for healthcare applications. Examples include wearable electronics for real‐time health monitoring and implantable devices for medical treatments. Therefore, this chapter focuses on the diverse and fascinating applications of TMDCs materials.

The unique physical properties and advantages of 2D materials such as MoS_2_ and WSe_2_ make them highly suitable for applications in piezoelectric electronic and optoelectronic devices. Their atomic thickness, mechanical flexibility, and high crystallinity allow them to endure substantial strain, which is essential for effective piezoelectric energy harvesting and sensing compare to the polymer such as PVDF and piezo‐ionic elastomer.^[^ ^134^, ^135^ ^]^ These materials exhibit significant piezoelectric coefficients, enabling the development of high‐performance PENGs that can convert mechanical energy into electrical energy efficiently.^[^ ^10^, ^136^, ^137^, ^138^ ^]^ The non‐centrosymmetric structure of 2D materials like MoS_2_ ensures strong piezoelectric effects, while their high carrier mobility and direct bandgap are ideal for FETs and optoelectronic applications such as photodetectors and photovoltaic devices.^[^ ^139^, ^140^ ^]^ Additionally, the ability to adjust bandgaps and electronic properties through strain or hetero structure enhances their versatility. For instance, the piezoelectric response of MoS_2_ can be significantly amplified by aligning its atomic orientation, which is essential for the sensitivity and efficiency of piezoelectric sensors. Moreover, WSe_2_ bilayers with turbostratic stacking demonstrate reliable piezoelectric properties and mechanical durability, making them appropriate for flexible and wearable electronics. These characteristics, combined with scalable synthesis methods like CVD, make 2D materials promising candidates for next‐generation electronic and optoelectronic devices, offering new opportunities in self‐powered sensors, energy harvesting, and advanced photonic applications. In addition, 2D materials are widely used as strain sensors in the healthcare field due to their softness and piezoelectric properties. By making flexible piezoelectric 2D materials into wearable flexible sensors, real‐time monitoring of human health can be achieved by sensing pulse or heartbeat. This non‐implanted flexible sensor avoids the risks brought by surgery. Even 2D piezoelectric materials can collect mechanical energy generated by human mechanical movement and convert it into electrical energy, thereby achieving self‐powered advantages. In addition, 2D materials have good biocompatibility and are widely used in the field of implantable biomedical. On the one hand researchers can use the piezoelectric effect of 2D materials to monitor the health level in the body. On the one hand, 2D materials have good piezoelectric catalytic effect. When external ultrasound is applied, 2D piezoelectric materials will collect vibration energy to cause deformation, thereby generating electric current to catalyze the production of reactive oxygen species (ROS). ROS can destroy the internal structure of cancer cells, thereby achieving safe, efficient and painless treatment.

### 4.1 PENGs

**Figure 7:**
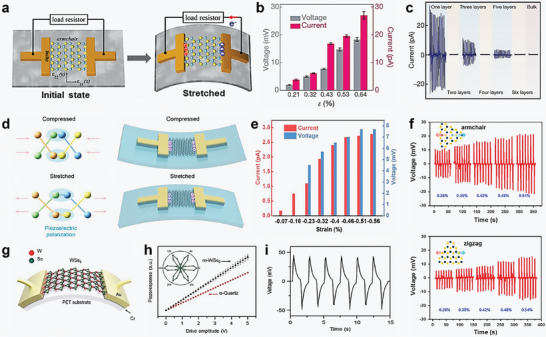
a,b) Schematic and piezoelectric output of PENG setup with monolayer MoS_2_ under strain, showing increased performance with higher strain. c) Current output of MoS_2_ flakes with different layer numbers, highlighting significant response in monolayer flakes. d,e) Piezoelectric polarization and outputs in BP under strain, demonstrating strong response. f) Voltage output comparison of armchair and zigzag orientations in MoS_2_, showing higher output in the armchair orientation. g) Schematic of turbostratic WSe_2_ bilayers on flexible PET substrate, showing reliable piezoelectric properties. h) Piezoelectric response of m‐WSe_2_ versus α‐quartz, indicating superior performance. i) Voltage output for flexible energy harvester based on turbostratic WSe_2_, demonstrating potential for continuous energy harvesting. Reproduced with permission,^[^ ^141^ ^]^ Copyright 2014, Springer Nature. Reproduced with permission,^[^ ^142^ ^]^ Copyright 2020, Wiley‐Blackwell. Reproduced with permission,^[^ ^143^ ^]^ Copyright 2016, Elsevier BV. Reproduced with permission,^[^ ^144^ ^]^ Copyright 2017, Wiley‐Blackwell.

Monolayer MoS_2_ exhibits significant potential in energy conversion and piezotronics due to its high crystalline quality and ability to withstand substantial strain. Wang et al. experimentally verified the piezoelectric properties of 2D MoS_2_ for the first time, discovering that odd‐layer MoS_2_ flakes generate oscillating piezoelectric voltage and current outputs during cyclic stretching and releasing, while even‐layer flakes do not exhibit this phenomenon (**Figure** **7**a).^[^ ^141^ ^]^ As shown in Figure 7b, a monolayer MoS_2_ under 0.53% strain can produce a voltage of 15 mV and a current of 20 pA, corresponding to a power density of 2 mW m^−^^2^ and a mechanical‐to‐electrical energy conversion efficiency of 5.08%. The study also demonstrated that piezoelectric output increases with decreasing thickness and reverses when the strain direction is rotated by 90°. Transport measurements revealed a significant piezoelectric effect in monolayer MoS_2_, but not in bilayers and bulk MoS_2_ (Figure 7c). These experimental results emphasize the potential of high‐quality monolayer MoS_2_ crystals for powering nanodevices, adaptive bioprobes, and tunable/stretchable electronic and optoelectronic devices. Furthermore, the study emphasizes the importance of crystal orientation in piezoelectric performance. In addition to crystal orientation, the atomic orientation within MoS_2_ significantly affects the performance of PENGs.

In addition to MoS_2_, other 2D materials have also been studied after being confirmed to have piezoelectric properties. Recent research by Ma et al. has highlighted the piezoelectric properties of multilayer black phosphorus (BP), a monoelemental 2D material with significant potential applications.^[^ ^142^ ^]^ Despite the typical absence of piezoelectricity in monoelemental materials due to lack of ionic polarization, BP exhibits piezoelectric behavior due to its non‐centrosymmetric lattice structure. The study reports in‐plane piezoelectricity along the armchair direction of BP, with current‐voltage measurements demonstrating a piezoelectric effect and cyclic compression of BP flakes showing an intrinsic current output of up to 4 pA under a compressive strain of −0.72% (Figure 7d). Figure 7e presents the current and voltage output as a function of applied strain, measured across a 1 GΩ load resistance, illustrating the piezo responses of BP. These findings are supported by theoretical calculations explaining piezoelectric polarization among P atoms. Additionally, the study reveals that the piezoelectric output and piezoelectric effects are more obvious along the armchair direction compared to the zigzag orientation. This research presents novel methods for the use of BP in biomechanical energy harvesting devices, electromechanical sensors, actuators, energy storage devices, transistors, and photodetectors. Moreover, it provides insights into achieving piezoelectricity in monoelemental materials without ionic polarization, highlighting the unique capabilities of BP for next‐generation electronic and optoelectronic applications.

Kim et al. focused on the impact of atomic orientation in CVD‐grown monolayer MoS_2_ on piezoelectric effects and their application in flexible PENGs.^[^ ^143^ ^]^ As shown in Figure 7f, PENGs with MoS_2_ in the armchair direction exhibited approximately twice the output power compared to those with MoS_2_ in the zigzag direction under the same strain of 0.48% and strain velocity of 70 mm ^−1^s. Additionally, lateral piezoresponse force microscopy was used to quantify the piezoelectric coefficient of monolayer MoS_2_, revealing that the *d*_11_ value is 3.78 pm V^−1^ in the armchair direction and 1.38 pm V^−1^ in the zigzag direction, clearly demonstrating the difference in piezoelectric effects due to varying atomic orientations. This study provides new insights for efficiently harvesting mechanical energy using novel flexible PENGs, showing promise for applications in high‐performance, low‐power devices and self‐powered piezoelectric electronic devices.

Moreover, the study by Kim et al. have shown that bilayers tungsten diselenide (WSe_2_) with turbostratic stacking exhibits significant piezoelectric properties, a feature absents in traditional Bernal‐stacked WSe_2_ bilayers.^[^ ^144^ ^]^ This is because the turbostratic stacking of CVD‐grown WSe_2_ monolayer increases the degrees in bilayers symmetry, leading to non‐centrosymmetry and thus strong piezoelectricity. In contrast, CVD‐grown WSe_2_ bilayers with Bernal stacking show weak piezoelectricity due to their centrosymmetric structure. The research by Lee et al. demonstrates that flexible piezoelectric WSe_2_ bilayers can withstand strains up to 0.95% and reliably harvest energy to power small devices like liquid crystal displays without external energy sources.^[^ ^144^ ^]^ The piezoelectric coefficients of monolayer and turbostratic bilayers WSe_2_ were measured to be 3.26 and 1.5 pm V^−1^, respectively (Figure 7g,h). This high output power, coupled with mechanical flexibility and stability, highlights the potential of turbostratic WSe_2_ in mechanical sensors, actuators, and energy sources for wearable and implantable electronics. This approach of turbostratic stacking can be extended to other 2D TMDCs materials to exploit their piezoelectric properties for various applications.

### 4.2 Piezotronic Devices

**Figure 8:**
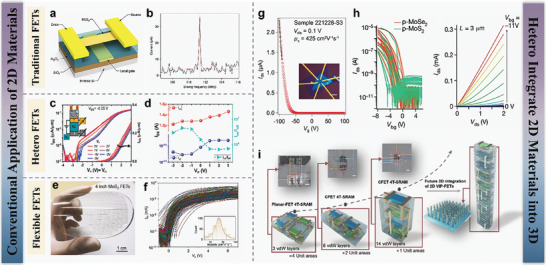
a) Schematic of a MoS_2_ transistor with a local gate configuration. b) Frequency response showing the device's high‐frequency operation. c) Transfer characteristics of the MoS_2_ transistor at various voltages, illustrating the gate‐tunable electronic properties, and d) modulation of on/off current ratios (I_on_/I_off_) and threshold voltage (V_th_) as a function of control voltage (V_P_). e) Image of a 4‐inch wafer containing multiple MoS_2_ FETs and f) mobility distribution of MoS_2_ transistors on the wafer, indicating consistent high performance. g) Output characteristics of p‐type MoSe_2_ and MoS_2_ transistors, with a highlighted high hole mobility of 425 cm^2^/V·s. h) Transfer characteristics of the transistors, showing excellent current modulation. i) Diagram of the scaling approach for integrating multiple layers of vdW materials into complementary FET (CFET) structures for vertical integration. Reproduced with permission,^[^ ^145^ ^]^ Copyright 2019, Springer Nature. Reproduced with permission,^[^ ^146^ ^]^ Copyright 2024, American Chemical Society. Reproduced with permission,^[^ ^147^ ^]^ Copyright 2023, Springer Nature. Reproduced with permission,^[^ ^36^ ^]^ Copyright 2024, Springer Nature.

Manzeli et al. have developed self‐sensing, tunable monolayer MoS_2_ nanoelectromechanical resonators (NEMS) with both piezoresistive and capacitive transduction mechanisms (**Figure** **8**a).^[^ ^145^ ^]^ These resonators exhibit resonant frequencies in the very high frequency (VHF) range, primarily defined by built‐in mechanical tension, and demonstrate high room‐temperature quality factors up to 300. Utilizing the piezoresistive effect, these devices achieve a gauge factor of ≈150, surpassing traditional capacitive transduction by threefold (Figure 8b). The study also explores nonlinear dynamic responses under high driving forces, focusing on the potential of MoS_2_‐based NEMS for applications in RF communications, mass, and force sensing. This work signifies a substantial leap in NEMS technology, providing insights into the mechanical effects at nanoscale dimensions. Varghese et al. at leverage the converse piezoelectric effect to achieve electrically controllable strain modulation in MoS_2_ FETs.^[^ ^146^ ^]^ Key innovations include the ability to switch the nature of the strain from compressive (0.23%) to tensile (0.14%) by reversing the polarity of the applied electrical bias, verified through Raman and photoluminescence spectroscopies. This method allows for precise and reversible tuning of device parameters, significantly enhancing the drain current (by 130x), on/off ratio (by 150x), and mobility (by 1.19x) (Figure 8c,d). The large strain gauge factors for both tensile (1056) and compressive (‐1498) strains demonstrate the high sensitivity and potential for integration with CMOS and MEMS technologies. This work represents a significant advancement in strain engineering, offering high thermal tolerance and compatibility with existing semiconductor technologies.

Tang et al. represents a significant advancement in the development of low‐power, flexible monolayer MoS_2_ integrated circuits (ICs).^[^ ^147^ ^]^ The authors introduced an ultra‐thin high‐κ dielectric/metal gate fabrication technique, enabling the creation of thin film transistors (TFTs) on both rigid and flexible substrates. These monolayer MoS_2_ TFTs demonstrated exceptional performance metrics, including a high electron mobility (>100 cm^2^/V·s), an on/off ratio exceeding 10⁸, a low subthreshold swing (≈60 mV dec^−1^), and ultra‐low leakage currents. These attributes are important for low‐power applications (Figure 8f). Moreover, the study achieved an innovative realization of large scale flexible ICs, capable of operating at voltages below 1 V, which is essential for portable, wearable, and implantable electronics. The study's success in integrating these advanced TFTs into flexible ICs paves the way for future developments in energy‐efficient flexible electronics.

This study by Guo et al. represents a significant breakthrough in the vertical 3D integration of 2D semiconductors, achieved through van der Waals (vdW) polarity engineering.^[^ ^36^ ^]^ The authors have successfully demonstrated the ability to reconfigure the carrier polarity of MoS_2_ from n‐type to p‐type by interfacing it with CrOCl, an antiferromagnetic insulator. This polarity inversion is attributed to a strong vdW interfacial coupling, resulting in exceptional room‐temperature hole mobilities of up to ≈425 cm^2^/V·s and on/off ratios reaching 10⁶ (Figure 8g,h). The devices exhibit impressive air stability, maintaining performance for over a year. The study also highlights the construction of vertically stacked complementary logic circuits, such as inverters, NAND gates, and SRAM cells, comprising up to 14 vdW layers (Figure 8i). These vertically integrated 2D complementary FETs (CFETs) demonstrate enhanced performance metrics, providing a promising route for future ultra‐high‐density 3D integrated circuits. The robustness and universality of this doping strategy across various 2D materials underline its potential for scalable semiconductor technologies. In addition to demonstrating high performance and stability, the research showcases the practical applicability of 2D material‐based CFETs in real‐world electronic applications. The ability to create complex logic circuits with multiple vdW layers without compromising device performance is a testament to the effectiveness of vdW polarity engineering. This advancement paves the way for the development of next‐generation electronic devices that are more compact, efficient, and capable of operating under diverse environmental conditions.

### 4.3 Piezo‐Phototronic Devices

**Figure 9:**
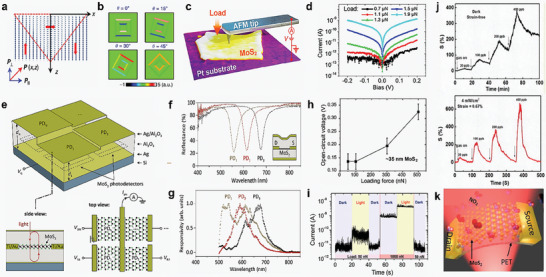
a) Polarization alignment in MoS_2_/ferroelectric heterostructure under varying angles. b) Simulated SHG intensity maps at different rotational angles. c) AFM tip applying load to MoS_2_ on Pt substrate to induce strain. d) *I–V* characteristics of MoS_2_ junction under varying mechanical loads showing resistance modulation. e) Optical cavity‐enhanced MoS_2_ photodetector array with adjustable peak responsivities. f) Open‐circuit voltage as a function of loading force, indicating enhanced photovoltaic response. g) Photocurrent response under dark and light conditions with varying mechanical loads. h) Reflectance spectra of the photodetectors showing distinct spectral features. i) Responsivity of photodetectors as a function of wavelength, highlighting spectral tunability. j) Flexible monolayer MoS_2_ NO_2_ gas sensor showing enhanced performance through strain‐induced piezo‐phototronic modulation of the Schottky barrier. k) Schematic of a flexible MoS_2_‐based NO_2_ gas sensor. Reproduced with permission,^[^ ^148^ ^]^ Copyright 2020, Springer Nature. Reproduced with permission,^[^ ^149^ ^]^ Copyright 2022, American Chemical Society. Reproduced with permission,^[^ ^150^ ^]^ Copyright 2023, Springer Nature. Reproduced with permission,^[^ ^153^ ^]^ Copyright 2019, Elsevier.

This study by Li et al. leverages the unique properties of vdW heterostructures and complex oxide interfaces to explore an unconventional nonlinear optical filtering effect.^[^ ^148^ ^]^ By combining monolayer MoS_2_ with a ferroelectric PbZr_0.2_Ti_0.8_O_3_ (PZT) thin film, the authors demonstrate a significant modulation in SHG signals. The results show that the SHG response at the heterointerface is either substantially enhanced or nearly quenched, depending on the chirality of the underlying ferroelectric domain wall (DW). This effect is purely mediated by the polar symmetry of the materials, rather than the commonly studied charge, spin, or lattice interactions. In their experiments, the authors illustrate the SHG mapping of the domain structures on PZT with and without the MoS_2_ top layer. The SHG intensity exhibits a robust dependence on the DW's orientation and chirality. Specifically, the horizontal DWs show alternating enhancement and suppression of SHG signals, attributed to the alignment of the polar axis of MoS_2_ with the in‐plane polarization of the PZT DWs (**Figure** **9**a,b). This study reveals a new method for designing nanoscale reconfigurable optical applications by exploiting the polar coupling at the heterointerfaces. The findings present way for developing electrically programmable optical filters using vdW materials and ferroelectric domain patterning, promising significant advancements in nanoscale photonic devices. The study by Chaudhary et al. explores the modulation of transport behavior in 2D MoS_2_ junctions under mechanical stress induced by an AFM tip (Figure 9c).^[^ ^149^ ^]^ They demonstrate that junction resistance can be reversibly tuned by up to four orders of magnitude by altering the tip‐induced force. Analysis of the stress‐induced evolution of the *I–V* characteristics indicates a combined effect of the tip‐induced strain and strain gradient on the energy barrier height and profile (Figure 9d). Additionally, the study reveals that the tip‐generated flexoelectric effect leads to a significant enhancement of the photovoltaic effect in MoS_2_ junctions (Figure 9h). Both strain and strain gradient are both important in modifying the MoS_2_ band structure, allowing for substantial resistance modulation (Figure 9i). The tip‐induced flexoelectric effect significantly impacts the photovoltaic effect in MoS_2_, demonstrating the potential for combined photomechanical tuning of resistance. This approach is applicable to any narrow band 2D semiconductor, enhancing their photovoltaic response. The fabrication of these 2D structures on flexible substrates, where a sizable strain and strain gradient can be induced, will facilitate the development of macroscopic optoelectronic devices with mechanically enhanced power.

In this study, Kwak et al. introduce a novel approach to optical spectroscopy through an in‐sensor computing paradigm using MoS_2_ photodetectors.^[^ ^150^ ^]^ The innovative device is designed to directly perform spectral regression or classification tasks at the physical level of photon detection, eliminating the need for external data processing. This is achieved by tailoring the spectral responsivity of the photodetectors to specific purposes. The authors utilized an ensemble of optical cavity‐enhanced MoS_2_ photodetectors with individually adjustable peak responsivities, enabling the device to analyze spectral mixtures efficiently. Figure 9e presents the design of the photodetector array with individually addressable photodetectors (PD1, PD2, PD3, PDN) capable of detecting different wavelengths. Figure 9e shows the reflectance spectra of the photodetectors, highlighting their ability to differentiate between different spectral components. Figure 9f,g displays the spectral responsivity of each photodetector, demonstrating the tailored response for specific wavelengths. The device's performance includes a high degree of tunability and efficiency, significantly advancing miniaturized and energy‐efficient optical sensing. The smart photodetector can approximate emission spectra of fluorescent dyes, perform real‐time analysis, and provide high sensitivity with low energy consumption. This approach not only reduces the complexity and size of conventional spectroscopic systems but also presents new possibilities for portable and wearable spectroscopic applications. Moreover, Piezo‐phototronic effect also can affect the performance of gas sensor and solar cells.^[^ ^151^, ^152^, ^153^ ^]^ As shown in Figure 9j,k, Guo et al. designed and fabricated a flexible monolayer MoS_2_‐based NO_2_ gas sensor and, for the first time, investigated the effect of the piezo‐phototronic effect on the performance of this monolayer MoS_2_‐based NO_2_ gas sensor. They found that the strain‐induced piezoelectric potential could effectively regulate electron and photogenerated carrier transport by enhancing the Schottky barrier, thereby further optimizing the sensor's performance.^[^ ^153^ ^]^

### 4.4 Wearable Piezoelectric Sensors for Healthcare

**Figure 10:**
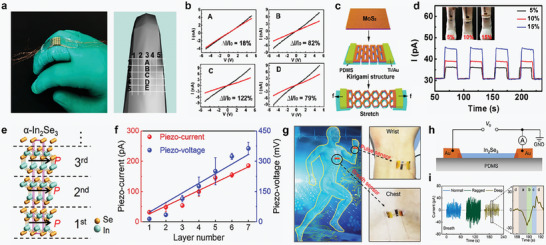
a) Photograph and 3D model of strain sensor array attached onto the midfinger to show position of devices A, B, C, D, E. b) *I*–*V* curves measured from the corresponding devices. c) Schematic illustration of the fabrication process of the MoS_2_/PDMS kirigami structure. d) Strain sensor based on the kirigami structure devices and the time‐dependent response of devices. e) Piezoelectric with the same polarization direction (black arrows) in a unit cell of α‐In_2_Se_3_. f) The piezoelectric current and voltage outputs as a function of α‐In_2_Se_3_ layer number. g) Real‐time monitoring of physiological signals (left). The piezotronic sensor based on few‐layer In_2_Se_3_ are attached on skin at the wrist (top right) and chest (bottom right) for monitoring arterial pulse and breath, respectively. h) The detailed structure of self‐powered piezotronic sensor based on a multilayer α‐In_2_Se_3_. i) Real‐time monitoring of breath signals. (a, breath in; b, breath gap; c, breath out; d, breath gap). Reproduced with permission,^[^ ^155^ ^]^ Copyright 2016, American Chemical Society. Reproduced with permission,^[^ ^156^ ^]^ Copyright 2018, American Chemical Society. Reproduced with permission,^[^ ^33^ ^]^ Copyright 2019, American Chemical Society. Reproduced with permission,^[^ ^32^ ^]^ Copyright 2019, American Chemical Society.

2D piezoelectric materials have acquired significant attention due to their high piezoelectricity per unit area and exceptional sensitivity, which enables the generation of high output even under micro‐vibrations.^[^ ^154^ ^]^ These properties make them highly suitable for sensor applications, and consequently, extensive research has been conducted in this area. With the growing demand for wearable devices, there has been a focus on developing sensors that can be attached directly to the skin. Feng et al. demonstrated a large‐area integrated strain sensor array utilizing In_2_Se_3_, a material characterized by a sizable bandgap and high stretchability, operating based on the piezoresistive effect.^[^ ^155^ ^]^ The authors synthesized high‐purity 2D In_2_Se_3_ using CVD and transferred In_2_Se_3_ onto a flexible PET substrate using a poly(methyl methacrylate) (PMMA)‐assisted transfer method to investigate its piezoresistive properties. Following the transfer, patterned Au electrodes were deposited, and large‐area strain sensor arrays were fabricated using Cu wiring (**Figure** **10**a). In Figure 10a, a 5 × 5 strain sensor array chip was attached to the back of the middle finger joint to test the functionality of the array on a conformable surface. The five devices along the center of the array (denoted as A, B, C, D, E in Figure 10a) successfully demonstrated spatial sensing capabilities. In Figure 10b, the spatial sensing results (ΔI/I0 ≈ 18% for A, ≈82% for B, ≈122% for C, ≈79% for D, ≈23% for E) were achieved even at a small spacing of 0.5 mm. These findings demonstrate that the sensor exhibits more than twice the sensitivity compared to conventional graphene and metal‐based strain sensors.

To enhance the sensitivity of strain sensors, a high mechanical strain tolerance in the material is essential. However, the limited mechanical strain of 2D materials poses challenges for their application in strain sensors. To address this limitation, Zheng et al. developed a strain sensor by introducing a kirigami structure to maximize the mechanical strain of MoS_2_ on a flexible substrate.^[^ ^156^ ^]^ Figure 10c illustrates the schematic of the kirigami‐structured MoS_2_ strain sensor, where the kirigami design enhances the in‐plane and out‐of‐plane strain, thereby improving the sensor's sensitivity. The authors used finite element method (FEM) simulations to demonstrate that the stretchability of MoS_2_ could be increased from the original 0.75% to ≈15%. Additionally, scanning electron microscopy (SEM) analysis revealed that when the strain exceeds 15%, damage occurs in MoS_2_ due to its limited strain. Based on these simulations and analyses, the authors evaluated the potential of the strain sensor by attaching it to the elbow of a humanoid robot and measuring the output in response to motion. As shown in Figure 10d, the current increased up to threefold as the strain increased, emphasizing that the mechanical limitations of 2D materials can be overcome through innovative structural designs. Another approach to enhancing the sensitivity of strain sensors lies in improving the intrinsic properties of the piezoelectric materials. Dai et al. proposed a self‐powered piezoelectric sensor using In_2_Se_3_, a material that overcomes the limitations of traditional 2D piezoelectric materials, where the piezoelectric properties typically diminish as the number of layers increases.^[^ ^33^ ^]^ In_2_Se_3_, with its AA stacking structure, maintains a consistent in‐plane polarization direction across layers like Figure 10e, leading to an increase in the piezoelectric stress coefficient (*e*_22_) as the number of layers increases. Remarkably, in the case of a 5‐layer structure, e_22_ was found to be four times greater than that of a monolayer in Figure 10f. Based on these findings, the authors fabricated a piezoelectric sensor by transferring In_2_Se_3_ onto a flexible PDMS substrate. The performance of the sensor was tested by attaching it to the wrist and chest, as illustrated in Figure 10g. Figure 10h shows the detailed structure of the piezoelectric sensor. The sensor, when attached to the wrist and chest, produced a higher frequency and larger current outputs (as shown in Figure 10i). This study demonstrates a significant advancement in the development of high‐performance piezoelectric sensors by leveraging the layer‐dependent piezoelectric properties of In_2_Se_3_, providing valuable insights for the future of strain sensor technology.

### 4.5 Piezoelectric Implants for Biomedical Therapy

Implanted 2D piezoelectric materials have emerged as a powerful tool in biomedical applications, particularly through their piezoelectric catalytic effects. These materials convert mechanical forces, such as ultrasound, into electrical stimuli, which in turn generate ROS or promote bioelectrical signaling.^[^ ^157^ ^]^ This process is being applied in critical areas like cancer treatment and nerve repair, where traditional methods often face limitations.^[^ ^157^ ^]^ By harnessing the unique properties of 2D piezoelectric materials, therapies can target hard‐to‐reach areas, enhance drug delivery, and stimulate cellular regeneration. In this part we explore three recent studies that demonstrate the versatility of piezoelectric materials in biomedical therapies. The first study uses a piezoelectric scaffold to stimulate nerve regeneration by enhancing Schwann cell activity and restoring bioelectrical signals. The second focuses on using piezoelectric catalytic nanomaterials to reduce tumor interstitial fluid pressure, improving the effectiveness of chemotherapy. The third demonstrates the use of sonodynamic therapy to enhance cancer treatment through ROS generation, facilitated by piezoelectric nanoribbons. These studies collectively highlight the transformative impact of 2D piezoelectric materials in advancing modern medical treatments.

**Figure 11:**
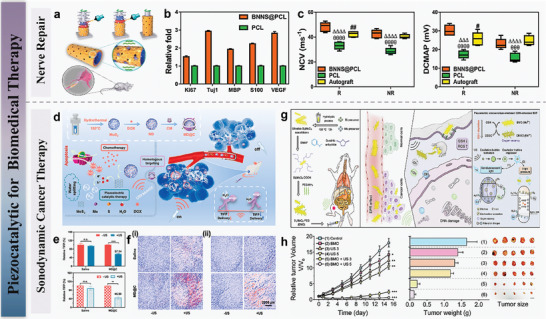
a) Schematic of nerve regeneration. b) Relative expression of neuronal markers (Ki67, Tuj1, MBP, S100, VEGF). c) Nerve conduction velocity (NCV) and distal compound muscle action potential (DCMAP) results comparing BNNS@PCL, PCL, and autograft groups. d) Schematic showing MoS₂‐mediated piezocatalysis for tumor therapy under ultrasound. e) Reduction of tumor interstitial fluid pressure (TIFP). f) Spot diffraction images of O_2_ content in tumors. g) Schematic of Bi_2_ MoO_6_ synthesis and SDT mechanism. h) Tumor volume and weight reduction across treatment groups in vivo. Reproduced with permission,^[^ ^158^ ^]^ Copyright 2021, Elsevier BV. Reproduced with permission,^[^ ^159^ ^]^ Copyright 2022, Elsevier BV. Reproduced with permission,^[^ ^160^ ^]^ Copyright 2021, Wiley‐Blackwell.

Qian et al. developed a boron nitride nanosheet (BNNS)‐functionalized polycaprolactone (PCL) scaffold for piezocatalytic neuronal repair, providing a new approach to peripheral nerve injuries. This study introduces the “microenvironment rebalance cocktail therapy,” which uses piezoelectric properties to stimulate neuronal activity and improve regeneration.^[^ ^158^ ^]^ The BNNS@PCL scaffold was engineered to generate piezoelectric stimulation under mechanical forces like ultrasound, increasing Schwann cell activity, enhancing bioelectrical signaling, and restoring energy metabolism in damaged neurons. The scaffold's elasticity, hydrophilicity, and biocompatibility provide an ideal microenvironment for nerve repair (**Figure** **11**a). In vitro studies showed that the Schwann cell protuberance length increased significantly, indicating superior cellular adhesion on the scaffold compared to the pure PCL scaffold. Moreover, Western blot analysis (Figure 11b) revealed a 2.9‐fold increase in the expression of the neural marker Tuj1 and a 1.9‐fold increase in myelin basic protein (MBP), which are critical for axonal growth and myelination. The scaffold also modulated oxidative stress by controlling ROS levels, which contributed to a balanced immune microenvironment and energy metabolism. In vivo experiments using a 15 mm sciatic nerve defect model demonstrated the scaffold's ability to regenerate nerves and restore bioelectrical signaling. As shown in Figure 11c, the nerve conduction velocity (NCV) in the BNNS@PCL group reached 48.2 ms^−1^, nearly matching the autograft group and significantly surpassing the PCL group (33.1 ms^−1^). Additionally, the distal compound motor action potential (DCMAP) was higher in the BNNS@PCL group, with a value of 30.2 mV, compared to 17.3 mV in the PCL group. The scaffold also enhanced vascularization, with Schwann cells expressing 2.8‐fold higher levels of vascular endothelial growth factor (VEGF), promoting neovascularization, which is essential for nutrient supply to regenerating nerves. This biocompatible scaffold demonstrated low systemic toxicity in vivo, providing a promising platform for clinical applications in peripheral nerve repair.

He et al. designed a novel tumor‐targeted piezoelectric catalytic therapy to address the significant issue of high tumor interstitial fluid pressure (TIFP), which prevents effective drug delivery in solid tumors^[^ ^159^ ^]^ Their approach involves the use of MoS_2_ nanoflowers (NFs) as a piezoelectric catalyst, encapsulated with a tumor cell membrane (CM) to form MD@C nanoparticles, loaded with doxorubicin (DOX). The MoS_2_ NFs' piezoelectric properties allow them to catalyze water splitting under ultrasound (US) stimulation, generating ROS and reducing the high TIFP in tumors. This is critical, as the high TIFP creates a reverse pressure gradient that impedes drug penetration. The US‐stimulated piezoelectric reaction not only facilitates drug delivery but also contributes to the destruction of tumor cells through ROS‐mediated damage(Figure 11d). In vivo experiments using U14 and PAN02 tumor‐bearing mice revealed that this method effectively reduced TIFP by 57.14% and 45.5%, respectively (Figure 11e). Furthermore, the combination of MD@C with US therapy showed remarkable tumor inhibition rates of 96.75% in U14 tumors and 99.21% in PAN02 tumors. The treatment significantly improved blood perfusion and oxygenation in the tumor, which enhanced the effectiveness of the loaded DOX. Another key observation was that, after treatment, the interstitial pressure drop allowed for a greater accumulation of blood‐derived oxygen and nutrients in the tumor (Figure 11f). This led to a marked increase in drug perfusion deep into the tumor tissue, improving the efficacy of chemotherapy. Additionally, biosafety evaluations indicated minimal systemic toxicity, as MD@C demonstrated excellent biocompatibility. Importantly, ROS generation from piezoelectric catalysis and DOX combined effectively, further boosting antitumor effects. The US‐triggered water splitting also resulted in the formation of hydroxyl radicals, which synergized with the chemotherapeutic action of DOX, enhancing tumor destruction while limiting systemic side effects. The study establishes MD@C as a promising, safe, and effective treatment strategy for solid tumors with high TIFP, providing a new avenue for improving the efficacy of cancer therapies.

Dong et al. introduced a novel sonodynamic therapy (SDT) method utilizing 2D piezoelectric Bi_2_MoO_6_ (BMO) nanoribbons (NRs) for glutathione (GSH)‐enhanced cancer treatment.^[^ ^160^ ^]^ The BMO NRs generate ROS under ultrasound (US) stimulation and simultaneously deplete GSH within the tumor microenvironment, addressing the issue of GSH‐mediated resistance to oxidative stress in cancer cells. By depleting GSH, the BMO nanoribbons enhance electron‐hole separation, which improves ROS production and thus boosts the therapeutic efficacy of SDT (Figure 11g). In this study, BMO NRs were functionalized with polyethylene glycol (PEG) to improve their biocompatibility and stability, resulting in GSH‐activated GBMO NRs. The piezoelectric properties of the GBMO NRs under US stimulation were confirmed, which demonstrates that the NRs can undergo mechanical strain to generate a piezoelectric field that accelerates ROS production. The generation of ROS by GBMO NRs led to enhanced oxidative stress in cancer cells, resulting in significant apoptosis. In vivo experiments, as seen in Figure 11h, showed that the treatment effectively reduced tumor volume, with a tumor inhibition rate reaching 96.6% in mice treated with GBMO NRs under US. This was significantly higher than treatments without ultrasound or GSH modulation. The high atomic number of bismuth in the NRs enabled excellent computed tomography (CT) imaging, which allowed for real‐time tracking of the treatment's progress. Furthermore, the treatment was well tolerated with minimal systemic toxicity, as confirmed by normal organ histology and stable body weights throughout the treatment course. This study presents a breakthrough in sonodynamic therapy by integrating piezoelectric nanomaterials with GSH modulation, offering a powerful platform for cancer therapy that combines treatment with diagnostic imaging, demonstrating both high efficacy and safety.

**Table** **1** below provides a summary of key 2D piezoelectric materials and their performance metrics across various applications, highlighting their potential in fields such as energy harvesting, piezotronic devices, piezo‐phototronic devices and sensor for healthcare and biomedical therapy.

**Table 1:** 2D piezoelectric materials in practical applications.

No.	Application	Material	Key Performance Metrics	Refs.
1	PENGs	Monolayer SnS and GeSe	Coefficients up to 16.1 pm V^−1^ for SnS and 10.2 pm V^−1^ for GeSe	[116, 117, 118]
2		GaP	Piezoelectric coefficient of ≈0.6 pm V^−1^	[121, 122, 123]
3		CIPS	Piezoelectric coefficient *d*_33_ of 17.4 pm V^−1^	[124, 125]
4		α‐In_2_Se_3_	Piezoelectric coefficient ranging from 0.34 pm V^−1^ in monolayer to 5.6 pm V^−1^ in bulk	[130, 131, 132]
5		Monolayer MoS_2_	Output voltage: 15 mV; current: 20 pA under 0.53% strain	[141]
6		Multilayer BP	Current output up to 4 pA under a compressive strain of −0.72%	[142]
7		Monolayer MoS_2_	Piezoelectric coefficient *d*_11_: 3.78 pm V^−1^ (armchair); 1.38 pm V^−1^ (zigzag)	[143]
8		Bilayers WSe_2_	Piezoelectric coefficient *d*_33_: 3.26 pm V^−1^ (monolayer); 1.5 pm V^−1^ (bilayers)	[144]
9	Piezotronic Devices	Monolayer MoS_2_	Resonant frequencies in VHF range; quality factors up to 300; gauge factor ≈150	[145]
10		MoS_2_	Drain current enhanced by 130×; on/off ratio increased by 150×; mobility improved by 1.19×	[146]
11		Monolayer MoS_2_	Electron mobility >100 cm^2^/V s; on/off ratio > 10^8^; subthreshold swing ≈60 mV dec^−1^; operating voltage <1 V	[147]
12		MoS₂ with CrOCl interface	Hole mobility up to ≈425 cm^2^/V s; on/off ratio up to 10^6^	[36]
13	Piezo‐phototronic Devices	Monolayer MoS_2_ on PZT	Significant modulation of SHG signals depending on ferroelectric DW chirality	[148]
14		MoS_2_	Tailored spectral responsivity; high sensitivity; direct spectral analysis at the detection level	[150]
15		Monolayer MoS_2_	Regulate electron and photogenerated carrier transport via Schottky barrier	[142, 151]
16	Wearable Piezoelectric Sensors for Healthcare	In_2_Se_3_	High sensitivity (ΔI/I₀ up to 122%); spatial resolution 0.5 mm; stretchability	[155]
17		Kirigami‐structured MoS_2_	Stretchability increased to ≈15%; current increases up to 3× under strain	[156]
18		AA stacking structured In_2_Se_3_	Piezoelectric current increases with layer number; monitoring of arterial pulse and breath; high sensitivity	[33]
19	Piezoelectric Implants for Biomedical Therapy	BNNS@PCL scaffold	Nerve conduction velocity up to 48.2 m ^−1^s; improved nerve regeneration; low systemic toxicity	[158]
20		MoS_2_ nanoflowers	Tumor interstitial fluid pressure reduced by up to 57%; tumor inhibition rates up to 99.21%; enhanced drug delivery	[159]
21		Bi_2_MoO_6_ nanoribbons	Tumor inhibition rate of 96.6%; GSH depletion enhances ROS generation; excellent CT imaging capability	[160]

## 5 Conclusion and Future Outlook

Research into 2D materials for piezotronics and piezo‐phototronics has made significant advancements, offering new opportunities for high‐performance electronic and optoelectronic devices. This review has highlighted the distinctive properties of 2D materials like MoS_2_ and WSe_2_ and their use in energy harvesting, flexible electronics, and sensors. These materials, characterized by their atomic‐scale thickness, exceptional flexibility, and tunable electronic structures, are poised to support next‐generation technologies. Nonetheless, the path to their widespread practical use requires addressing several crucial technical challenges.

One primary challenge is the scalable and controlled synthesis of high‐quality 2D materials. Current methods such as CVD show promise but encounter hurdles when scaled for industrial applications, including high production costs, complex synthesis protocols, and difficulties in achieving uniformity and reproducibility. To address these challenges, developing innovative synthesis methods like roll‐to‐roll CVD, metal‐organic CVD, and low‐temperature solution‐based techniques will be essential. Cost reduction through more affordable precursors, process optimization, and material recycling strategies will also be critical for mass production. Additionally, ensuring 2D material stability in practical applications is vital, as they can be prone to oxidation and environmental degradation. Implementing surface passivation, protective coatings, and composite material strategies can enhance their durability and environmental resistance.″

Different types of 2D materials come with unique benefits and challenges that require targeted solutions. For instance, TMDCs like MoS_2_ and WSe_2_, despite their superior electronic and mechanical properties, face difficulties in achieving large‐area uniform growth and precise layer control. Refining growth parameters, utilizing catalysts, and incorporating substrate engineering will be key to improving the quality of these materials. BP, known for its tunable bandgap and high carrier mobility, poses challenges due to its susceptibility to oxidative degradation. Protective surface layers, chemical modifications, or encapsulation within inert environments can significantly enhance their stability. Addressing these material‐specific challenges will necessitate interdisciplinary collaboration across materials science, chemical engineering, and biomedical engineering fields.

Overcoming these challenges is vital for 2D materials to see broader applications. While substantial progress has been made in their use in flexible sensors, energy harvesters, and biomedical implants, further efforts are needed. Application‐driven material design can customize properties for specific needs; for example, In_2_Se_3_‐based wearable sensors offer real‐time physiological monitoring, while MoS_2_ nanoflowers have shown promise in cancer treatment. Advanced characterization techniques like in‐situ microscopy and synchrotron radiation can provide deeper insights into the behavior of 2D materials under operational conditions, guiding modifications and performance improvements. Establishing standardized testing for assessing performance and reliability across different environments will create a foundation for commercial use. Prioritizing long‐term stability tests and developing low‐cost, scalable manufacturing processes such as solution‐phase synthesis for printed electronics can lower costs and enhance scalability. Collaborative efforts among various scientific disciplines will continue to be essential for innovation in 2D materials and their device applications. For instance, integrating 2D materials with biocompatible polymers has already led to implantable medical devices that promote nerve regeneration. Ensuring the biocompatibility and biodegradability of these materials through comprehensive evaluations is a necessary step for safe medical application. The flexibility and tunable electronic properties of 2D materials present enormous potential for developing next‐generation implantable electronic devices capable of monitoring physiological functions or providing targeted therapies. Addressing these challenges through focused research and practical applications will bridge the gap between theoretical innovation and real‐world implementation, driving the use of 2D piezoelectric materials in electronics, optoelectronics, and biomedical devices.

## Conflict of Interest

The authors declare no conflict of interest.
